# We are on the right track for mantle cell lymphoma

**DOI:** 10.1111/bjh.20259

**Published:** 2025-07-06

**Authors:** Alessia Moioli, Carlo Visco

**Affiliations:** ^1^ Centro Ricerche Cliniche s.r.l. Verona Italy; ^2^ Department of Engineering for Innovation Medicine, Section of Hematology AOUI VR, University of Verona Verona Italy

**Keywords:** bendamustine, epidemiology, mantle cell lymphoma, survival, Sympatico, Triangle

## Abstract

Commentary on: Alzahrani et al. Therapeutic advances and gradual improvement in overall survival in mantle cell lymphoma over 4 decades. Br J Haematol 2025; 207:591‐596.

In their paper, Alzahrani et al. report a comprehensive population‐based analysis of mantle cell lymphoma (MCL) patients treated over four decades, reporting the impact of the evolution of first‐line therapies on patient outcomes.^1^ By stratifying patients across distinct therapeutic eras—defined by major shifts in front‐line standards—the authors provide a real‐world perspective on long‐term outcomes, clearly showing the persistent survival disadvantage when compared to age‐ and sex‐matched individuals in the general population.

MCL is an aggressive subtype of non‐Hodgkin lymphoma, characterized by a heterogeneous clinical course and a high propensity for relapse. Over the past four decades, patient survival has significantly improved, initially due to the introduction of intensified treatment strategies. These included high‐dose cytarabine‐ and rituximab‐based induction regimens, followed by autologous stem cell transplantation and maintenance therapy in younger and fit patients.^2^, ^3^ For older patients, the introduction of bendamustine plus rituximab proved to be an effective and well‐tolerated option.^4^ However, the introduction of Bruton's tyrosine kinase inhibitors (BTKi) was the real breaking point with the past, above all for their ability to provide an effective therapeutic option in patients who were relapsed or refractory to standard inductions, at any age. For about 10 years from now, prospective and retrospective observations, including comparison with alternative available options, have established covalent‐BTKi (i.e. ibrutinib, acalabrutinib and zanubrutinib depending on the prescribing possibilities in the geographical areas of the world) as the preferable choice at first relapse or progression both in early‐ or late‐relapsing patients.^5^

In this context, the current study provides meaningful insights by analysing survival trends across three treatment eras: Era 1 (cytotoxic chemotherapy only), Era 2 (addition of rituximab and autologous stem cell transplantation) and Era 3 (incorporation of bendamustine and targeted agents in the relapsed/refractory setting). The most substantial survival gains were observed between Era 1 and Eras 2 and 3, underscoring the transformative impact of immunochemotherapy and consolidation strategies. The more modest improvements seen from Era 2 to Era 3 likely reflect the still‐developing role of targeted therapies in real‐world practice. As expected, patients receiving BTKi had significantly better outcomes than those who did not receive BTKi. Evidence suggests that ibrutinib is more effective when used at first relapse rather than later lines, supporting the idea that earlier integration of targeted therapies can significantly improve prognosis. Age‐stratified analyses further revealed the distinct patterns of benefit: younger patients (<65 years) derived the greatest survival gains between Era 1 and Era 2, likely driven by high‐dose chemotherapy, autologous stem cell transplantation and maintenance therapy. In contrast, older patients (≥65 years) showed more modest but meaningful improvements primarily in Era 3, likely reflecting the benefit of bendamustine‐based regimens better suited for less fit populations. Of note, after adjusting for biological and clinical factors such as Mantle Cell Lymphoma International Prognostic Index (MIPI) and morphological variants, the apparent survival benefit attributed to newer therapies was attenuated, thus reflecting the clinically and biologically heterogeneous nature of MCL.

Although recent population‐based studies have demonstrated improvements in survival outcomes for MCL, they were often limited by retrospective designs and incomplete clinical, molecular or treatment data. Follow‐up periods tended to be relatively short, especially for more recently diagnosed patients, and few studies provided direct comparisons with age‐ and sex‐matched individuals from the general population.^6^, ^7^ We are all aware that clinical trials may not fully reflect outcomes in the broader, more heterogeneous patient population seen in routine clinical practice, where access to novel therapies and intensive monitoring is frequently restricted. Instead, long‐term population‐based studies like the one from Alzahrani et al., which spanned over four decades and included almost all patients within a defined region, provided invaluable real‐world insights, shedding light on both therapeutic progress and the persistent survival challenges faced by patients with MCL. Importantly, despite the advances described in the three therapeutic eras, a significant survival gap remained when comparing MCL patients to age‐ and sex‐matched individuals from the general population. This gap is especially pronounced outside clinical trials and specialized referral centres, where access to treatment, implementation of risk‐adapted strategies and intensity of patient monitoring can vary widely.

That said, the current era of treatment in MCL is bringing substantial and ongoing innovations over time, which will reflect into further improvements in the survival of our patients (Figure 1). Most of the studies that are integrating BTKi into front‐line treatment, either in younger or older patients, have mature results indicating a significant further step towards survival improvement.^8^, ^9^ In the relapsed/refractory setting, results from the SYMPATICO study showed that the combination of ibrutinib plus venetoclax confers superior efficacy compared with single‐agent ibrutinib in patients with relapsed or refractory MCL. The analysis represented the first randomised trial to demonstrate improved progression‐free survival in patients with relapsed or refractory MCL and *TP53* mutations.^10^

**FIGURE 1:**
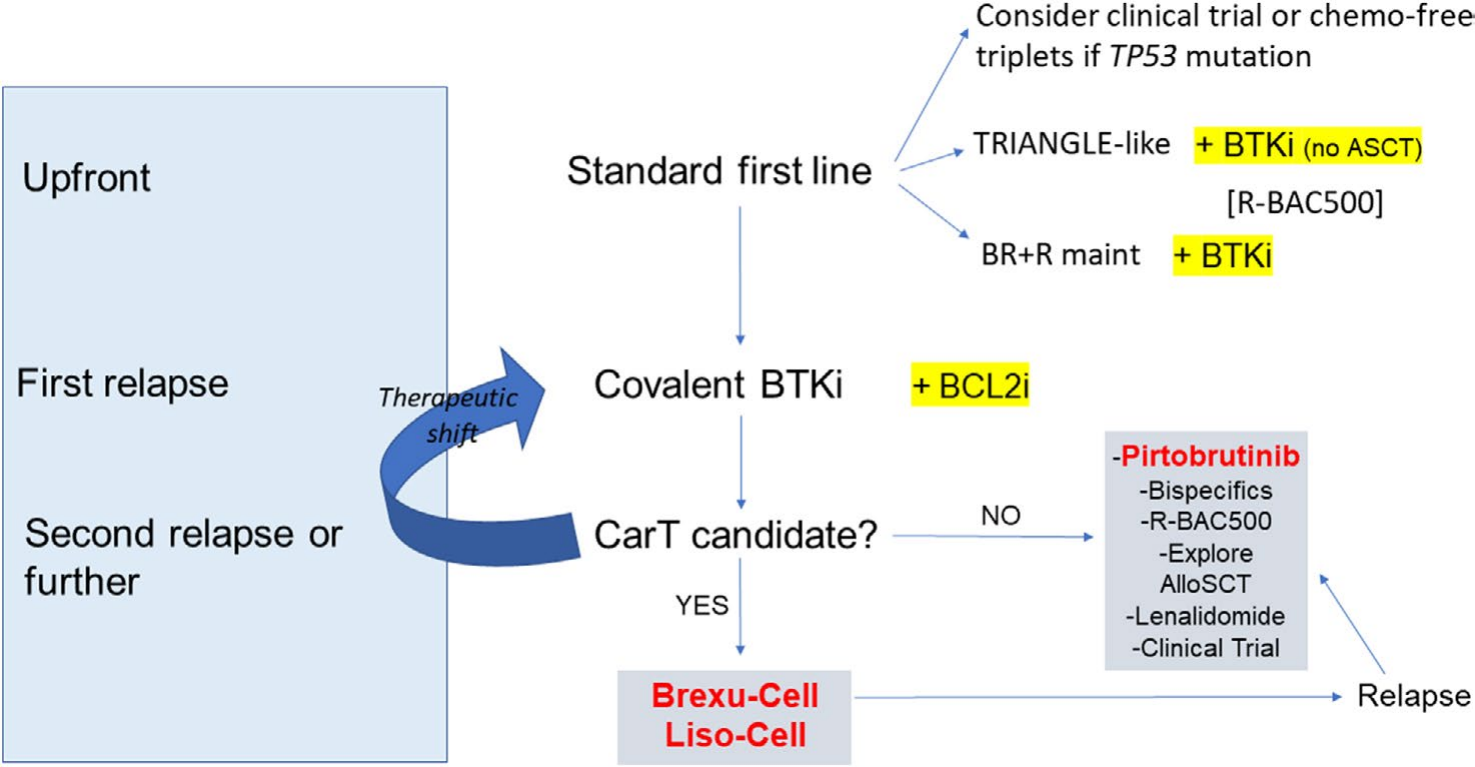
General example of therapeutic algorithm.

On top of that we must recognize the ever‐increasing role non‐covalent BTKi (i.e. pirtobrutinib), chimeric antigen receptor (CAR)‐T‐cell therapy (i.e. brexucaptagen autoleucel and lisocabtagene maraleucel) and bispecific antibodies (i.e. glofitamab) have in the second line of treatment or beyond, with BTK‐degraders, next‐generation B cell lymphoma 2 (BCL‐2) inhibitors and several other molecules that are investigated in phase 1–2 trials (e.g. NCT05471843, NCT06742996, NCT06839053, NCT05544019).

In conclusion, while we are significantly improving the overall survival of patients with MCL, we have to recognize that this lymphoma subtype still remains a difficult‐to‐treat malignancy, with many patients that still have limited survival. Along with the integration of newer strategies and drugs, global future efforts should focus on refining biological risk assessment, ensuring prompt use of combination treatments and adopting increasingly personalized therapeutic approaches (Figure 1). Overall, we need to congratulate colleagues from British Columbia for putting together these data; showing major increases in effective therapies for MCL, but an expected loss of life expectancy compared to a matched general population. Still, we believe that we are on the right track for MCL patients. Our next primary goal will be to ensure that everyone, everywhere in the world and all categories of patients with MCL will invariably receive the best available care.

## AUTHOR CONTRIBUTIONS

Alessia Moioli and Carlo Visco wrote the paper and revised it critically. Both authors approved the final version.
